# The Reliability and Validity of Gluteal Endurance Measures (GEMs)

**DOI:** 10.26603/001c.29592

**Published:** 2021-12-02

**Authors:** B J Lehecka, Barbara S Smith, Todd Rundell, Thomas A Cappaert, Nils A Hakansson

**Affiliations:** 1 Physical Therapy Wichita State University; 2 Health Sciences Rocky Mountain University of Health Professions; 3 Biomedical Engineering Wichita State University

**Keywords:** gluteals, endurance, electromyography, movement system

## Abstract

**Background:**

The gluteals have unique morphology related to muscle endurance, including moderate fiber sizes and a majority of Type I endurance fibers. Evidence suggests gluteal endurance is related to low back pain, running kinematics, balance, posture, and more. However, reliable and valid measures specific to gluteal endurance are lacking in the literature.

**Hypothesis/Purpose:**

The purpose of this study was to examine the intra- and inter-rater reliability of two gluteal endurance measures (GEMs) for clinical use. It also aimed to examine validity for the two measures by using electromyography (EMG), recording reasons for task failure, and analyzing differences between demographic groups.

**Study Design:**

Cross-Sectional

**Methods:**

Sixty-eight males and females with and without recurrent low back pain aged 18-35 years were recruited from a university population. Electromyography electrodes were placed on subjects’ gluteus maximus and gluteus medius, and each subject performed three trials of GEM-A (abduction endurance) and GEM-B (bridging endurance). Hold times, EMG median frequency (MF) data, and subjective reasons for task failure were analyzed.

**Results:**

Both GEMs demonstrated high intra-rater reliability (ICC = 0.87-0.94) and inter-rater reliability (ICC = 0.99). Mean hold times were 104.83 ± 34.11 seconds for GEM-A (abduction endurance) and 81.03 ± 24.79 seconds for GEM-B (bridging endurance). No statistically significant difference was found between subjects with and without recurrent LBP. Median frequency data validated the onset of gluteal fatigue during both measures. Posterolateral hip (gluteal) fatigue was reported as the primary reason for task failure in 93% and 86% of subjects for GEM-A and GEM-B, respectively.

**Conclusion:**

This seminal study of GEM-A (abduction endurance) and GEM-B (bridging endurance) found both measures to be reliable and valid measures of gluteal endurance. Further examination of the GEMs in samples with different types of LBP or hip pain is recommended.

**Level of Evidence:**

3

## INTRODUCTION

In 1999, McGill et al.^1^ published a study of clinical targets and reliabilities for submaximal isometric trunk endurance exercises in a healthy university population. These exercises included isometric trunk flexion, trunk extension, and side bridge exercises held to fatigue. Since that seminal study, those endurance measures have been used to determine endurance deficits in several specific populations, notably subjects with varied classifications of low back pain.^2–9^ McGill and others recognized the need to measure muscle endurance as a construct separate from muscle strength, a distinction especially evident in core muscles including the gluteals.^10–12^

The gluteals have unique morphology related to muscle endurance. The gluteus medius is composed of about 58% Type I fibers, fibers with high oxidative capacity oriented for endurance.^13^ The gluteus maximus is composed of about 52-68% Type I fibers.^13,14^ Most muscles display a combination of fiber types, but the gluteals have a larger percentage of Type I fibers than several other lower limb muscles including the rectus femoris (38%), vastus lateralis (42%), and gastrocnemius (48%).^13^ In another cadaveric study, the gluteals were shown to have a moderate fiber size, neither small nor large, again reflecting their purpose for both strength and endurance activities.^15,16^

Time to failure of the side bridge is frequently used to assess lateral trunk endurance. It has been inversely correlated to the development of low back pain (LBP) during standing,^6^ LBP in tennis players,^3^ LBP in female university dance students,^9^ work-related musculoskeletal disorders in manual lifting workers,^4^ peak internal rotation during running in female runners,^7^ and static balance during single-limb stance in male university students.^2^ The side bridge has been shown to elicit high gluteus medius electromyographic (EMG) activity (74% MVIC) and low gluteus maximus activity (21% MVIC).^17^ At the same time, it elicits considerable activity from the external oblique (69% MVIC) and other trunk muscles.^17^ The adductor muscles also likely assist in keeping the pelvis lifted from the ground, although their contribution has not been measured via EMG. In a study by Greene et al.,^18^ subjects performing the side bridge as a test of endurance were asked their reasons for task failure and less than 50% cited side or hip fatigue or pain as the primary cause. Over 40% reported upper extremity fatigue or pain as their reason for failure during the side bridge. This indicates that although the side bridge elicits high EMG activity of the gluteus medius, it has limited specificity to gluteal endurance.

The supine bridge, a common measure of posterior trunk endurance, has been shown to elicit both gluteus maximus and gluteus medius EMG activity, but at low levels (25% and 28% MVIC, respectively).^17^ Another limitation of the supine bridge as a measure of gluteal endurance is its use of bilateral lower extremities. This bilateral activity offers little information for determining unilateral endurance deficits. Time to failure of the supine bridge has, however, been correlated to chronic mechanical back pain, and pain and disability in patients with lumbar spondylolisthesis.^19,20^

The EMG activity of a unilateral bridge with the knee flexed to 90° has been examined in multiple studies, and ranges from 40-51% MVIC for the gluteus maximus and 47-57% MVIC for the gluteus medius.^17,21–24^ The unilateral bridge with the knee flexed to 90° has been examined as an endurance task in one study.^25^ However, it was performed only by a relatively small sample size (n=20) of healthy subjects and no reliability data were calculated. Also, the unilateral bridge with the knee flexed to 90° has been shown to elicit higher hamstring activity (up to 75% MVIC) than gluteal activity, causing the hamstrings to likely be the limiting muscle during the unilateral bridge, instead of the gluteals.^24^ The side and supine bridges have value as endurance measures; however, treatment for endurance deficits could be better targeted if the endurance measures implemented were targeted to a specific muscle group rather than several muscle groups.

Gluteal endurance appears related to several classifications of low back pain,^3,6,9,19,20,26–28^ work-related musculoskeletal disorders,^4^ running kinematics,^7^ balance,^2^ and pelvic posture.^29^ Given the morphology of gluteal muscles related to endurance, the lack of clinical endurance measures specific to the gluteals, and the link between various pathologies or impairments and gluteal or trunk endurance, the purpose of this study was to examine the intra- and inter-rater reliability of two gluteal endurance measures (GEMs) for clinical use. It also aimed to examine validity for the two measures by using electromyography (EMG), recording reasons for task failure, and analyzing differences between demographic groups. It was hypothesized that GEM scores would demonstrate high intra- and inter-rater reliability, demonstrate highly reliable and negative median frequencies (demonstrating gluteal fatigue), and have lower standard errors of measurement (SEMs) than related measures. It was also hypothesized that the majority of subjective reasons for task failure would be posterolateral hip (gluteal) fatigue for both GEMs, and that subjects with recurrent LBP would have lower GEM scores than subjects without recurrent LBP.

## METHODS

Male and female students between the ages of 18 and 35 years (23 male, 45 female; average age 22.78 ± 2.47 years; average BMI 23.39 ± 12.68 kg/m; average activity level 147.01 ± 111.03 minutes of moderate aerobic activity and 90.51 ± 81.75 minutes of vigorous aerobic activity per week) were recruited with emails and word-of-mouth from a local university. Both undergraduate and graduate students in health-related fields volunteered. Exclusion criteria were adapted from a similar study of muscle endurance by Shellenberg et al.^19^: a history of angina or emphysema; diagnosed spinal or hip abnormality; abdominal, back, or lower extremity surgery within the past year; or pregnancy. Subjects recruited with a history of recurrent LBP denied pain on the day of testing. Estimating a prevalence of recurrent LBP of 24% based on a study by Stanton et al,^30^ 68 subjects were recruited. Of those 68 subjects, 51 presented without recurrent LBP (16 male, 35 female) while 17 reported recurrent LBP (6 male, 11 female). This sample size, accommodating for a 10% drop-out rate, was based on the number needed to detect a large effect size of 0.8 with a study power of 0.8 and an alpha level of 0.05.^31^

The study was conducted in a biomechanics laboratory at a local university. Institutional Review Board approval was acquired prior to subject recruitment. Subjects arrived in exercise attire and first completed a consent form, current health history questionnaire, and activity and demographics questionnaire. Age, height, weight, sex, leg dominance, and average weekly aerobic activity were recorded on the activity and demographics questionnaire. Subjects were introduced to the testing procedures, including the specifics of the GEMs, familiarization trial, electrode placement, and rest periods.

Surface EMG electrodes were used to record muscle activity as the subjects performed the GEMs. Before electrode placement, the skin over the gluteals was cleaned and abraded with alcohol wipes by the subjects following instruction of the procedure. Surface electrodes were placed by subjects in a private room following researcher instruction, then appropriate placement was confirmed by the lead researcher via palpation over the subjects’ clothing. Bi-polar electrodes were placed on the gluteus maximus and gluteus medius of the dominant leg as determined by which leg would be used to kick a ball. Electrode placement was based on related studies and standard practice.^17,24,32^ A ground electrode was placed over the ipsilateral anterior superior iliac spine. The electrode to record gluteus maximus activity was placed midway between the lateral border of the sacrum and the posterosuperior edge of the greater trochanter on the muscle belly. The electrode to record gluteus medius activity was placed anterosuperior to the gluteus maximus, inferior to the lateral aspect of the iliac crest on a line towards the greater trochanter on the muscle belly. Surface EMG data were collected at 3000 Hz using a Noraxon TeleMyo 2400T GT (Noraxon, Scottsdale, AZ).

**Figure 1.**
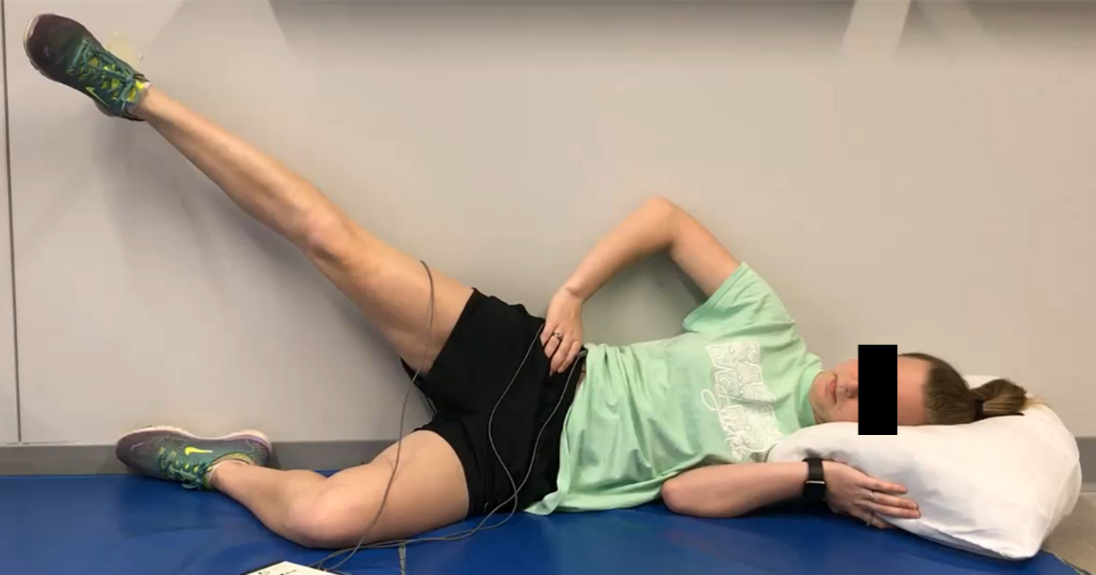
Gluteal endurance measure A (GEM-A) – abduction endurance

**Figure 2.**
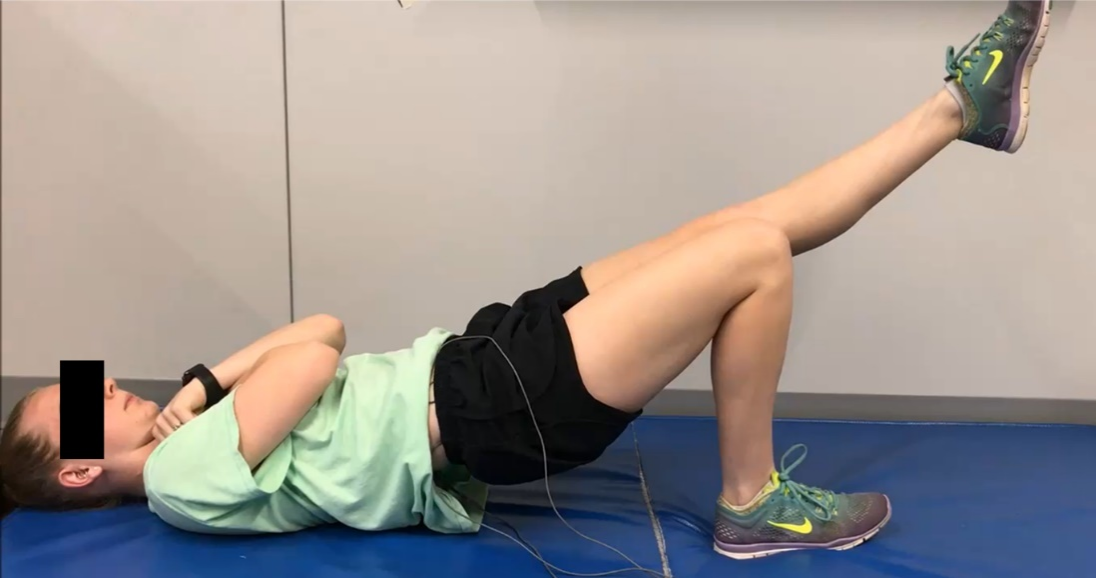
Gluteal endurance measure B (GEM-B) – bridging endurance

The order of GEM-A (Figure 1) and GEM-B (Figure 2) performance was determined via coin flip. Descriptions of GEM-A and GEM-B are provided in Table 1. The GEMs were only tested in the dominant lower extremity as there was no significant difference in hold time or MF slopes between sides in studies of similar core endurance tasks.^1,33,34^ Following familiarization of each position, three trials of each of the two GEMs were performed with 10 minutes of rest between each trial. Rest durations for similar endurance tasks in other studies vary, including a minimum of five minutes between repetitions and a 1:4 work-to-rest ratio.^1,19^ Preliminary testing revealed noticeable decreases in GEM scores between the first and third repetitions of each measure when five minutes of rest were provided, but no significant differences existed between trials when 10 minutes of rest were provided between repetitions. The lead researcher was blind to the GEM scores (times to task failure recorded in seconds) as a lab technician started and stopped the EMG software upon verbal direction while the lead researcher monitored each subject. Subjects were asked “Why did you stop the test?” immediately following each trial, and their reasons for task failure were recorded in an electronic spreadsheet by the lead researcher. This question was identical to the question used in a study of side bridge variations by Greene et al.^18^

The minimum number of subjects needed for reliability analysis was determined for a desired power of 0.8, an alpha level of 0.05, three repeated measures for intra-rater reliability, and three repeated measures for inter-rater reliability. The anticipated intra-rater reliability of GEM scores (recorded in seconds) was 0.9 based on the largest study of the side bridge’s reliability.^1^ Using a method described by Walter et al. and the aforementioned values, it was determined that at least 13 subjects were needed for intra- and inter-rater reliability analysis.^35^ Therefore, 13 healthy subjects and 13 subjects with recurrent LBP were video-recorded performing the GEMs. Recurrent LBP was defined as two or more episodes of LBP lasting more than 24 hours with a numeric pain rating scale (NPRS) score of more than 2/10 within the past year, and with at least a 30-day pain-free period between episodes.^36^ These video recordings were taken consecutively from the start of the study until 13 subjects from each group were recorded. A second researcher used the recordings to determine time to task failure of the GEMs. These data were used for inter-rater reliability analysis. The recordings were muted to blind the second researcher from the verbal direction used between the lead researcher and lab technician. The second researcher was blinded to all subject data besides the muted recording, including group assignment and time to task failure recorded by the lead researcher on the EMG software.

**Table 1.** Descriptions of gluteal endurance measures (GEMs)

GEM Title	GEM Description
GEM-A (abduction endurance)	The subject is sidelying with the back parallel to and lightly touching a wall for spatial reference. The hip and knee of the top lower extremity (the extremity being tested) are in 0° of flexion and rotation, resting on the bottom lower extremity. The bottom extremity’s knee is flexed to 90°, and its hip is flexed near 45° to allow the sole of the foot to rest on the posterior wall. Shoes are worn. The hand of the top arm rests lightly on the top iliac crest for pelvic monitoring. The bottom arm rests in a relaxed, comfortable position, and the subject’s head rests on a standard pillow with the trunk in a neutral position. The subject’s uppermost lower extremity is passively abducted by the tester to 30° as measured by an inclinometer. The tester then releases hold of the extremity and instructs the subject to actively maintain the hip in 30° of abduction as long as possible. The tester is allowed to give cues to the subject during testing to re-achieve correct positioning; however, no motivational cues are given. The tester monitors the subject’s position using a tape marker placed on the wall near the subject’s raised heel until the test ends. The subject is not told or able to see the time elapsed during until all testing is complete. The test ends when the tester observes an estimated loss of over 25% of the starting position height for more than three seconds, or the tested limb contacts the testing surface. The time to task failure is recorded.
GEM-B (bridging endurance)	The subject is hooklying with the arms across the chest. The tested extremity’s knee is flexed to 135° or as near to that position as able. The feet are placed shoulder-width apart. Shoes are worn. The non-tested extremity’s knee is extended to 0° of flexion, and its thigh is held parallel to the tested extremity’s thigh throughout the test by the subject. The subject is instructed to actively extend the tested extremity’s hip to 0° of flexion (or nearest to this position as possible) as measured by the tester using a goniometer. The subject is instructed to maintain the hip in 0° of flexion as long as possible. The tester is allowed to give cues to the subject during testing to re-achieve correct positioning; however, no motivational cues are given. The tester monitors the subject’s position until the test ends. The subject is not told or able to see the time elapsed until all testing is complete. The test ends when the tester observes an estimated loss of over 25% of the starting position height for more than three seconds, or the pelvis contacts the testing surface. The time to task failure is recorded.

Surface EMG data were collected at 3000 Hz, rectified, and filtered using a 4^th^ order Butterworth filter with a pass-band between 5 and 500 Hz. Median frequency was calculated within a one-second moving window every 100 milliseconds (ms). Time to task failure of the GEMs was recorded for all subjects using the EMG software program. The lead researcher used verbal “start” and “stop” directions while a lab technician simultaneously started and stopped data collection within the program. While watching recorded video, a second researcher used a stopwatch to record time to task failure of all GEM trials for the 26 subjects used for inter-rater reliability analysis (13 healthy subjects, and 13 with recurrent LBP). Subjective reasons for task failure were recorded after each trial on an electronic spreadsheet. Data were processed using custom written code (Matlab, The Mathworks, Natick, MA). The MF slope values were recorded in hertz per second (Hz/s). The times to task failure were recorded in seconds (s). SPSS v23.0 (SPSS Inc, Chicago, IL) was used for data analysis.

Intraclass correlation coefficients (ICC) were used to determine intra- and inter-rater reliability of the GEM scores and MF slopes. Traditional formulas were used to calculate standard errors of measurement (SEM) and minimal detectable changes (MDC) for GEM scores.^37^ Pearson’s correlation coefficients were used to determine the correlation between GEM scores, MF slopes, and body mass index (BMI). Independent t-tests were used to determine significant differences between subjects using the presence of recurrent LBP, sex, and BMI as independent variables on the dependent variables of GEM scores and MF slopes. Means and standard deviations were calculated for GEM scores and MF slopes. Frequency values were calculated for subjective reasons for task failure after categorizing each subject response as posterolateral hip (gluteal) fatigue, contralateral posterolateral hip fatigue, low back (erector spinae) fatigue, anterior thigh (quadriceps) fatigue, posterior thigh (hamstring) fatigue, or lower leg (triceps surae) fatigue. No subject reported pain as the primary reason for task failure, and all responses fell in one of the aforementioned six categories.

## RESULTS

Sixty-eight subjects were measured. Data from 66 subjects were included, 49 healthy subjects and 17 subjects with recurrent LBP. Data from two subjects (females without recurrent LBP) were excluded due to faulty data from the EMG leads over the gluteals. Descriptive statistics for GEM scores are displayed in Table 2. Results include data from 22 males and 44 females with an average BMI of 23.39 ± 12.68 kg/m and age of 22.78 ± 2.47 years. Times from both GEMs demonstrated high intra- and inter-rater reliability (ICC = 0.87-0.94 and ICC = 0.99, respectively). The MF slopes for both gluteal muscles also demonstrated high reliability for each GEM (ICC = 0.74-0.82 for GEM-A; ICC = 0.70-0.83 for GEM-B). The standard errors of measurement (SEM) for GEM-A and GEM-B scores were 8.36 and 8.94 seconds, respectively. These SEM values equate to minimal detectable changes (MDC) of 23.17 and 24.78 seconds for GEM-A and GEM-B, respectively.

**Table 2.** Descriptive statistics for GEM scores (hold times)

Measure	Subject description	Mean ± SD (*s*)	N
GEM-A (abduction endurance)	All subjects	104.83 ± 34.11	66
	Healthy subjects	105.14 ± 36.37	49
	Subjects with recurrent LBP	103.93 ± 27.53	17
	Males	92.46 ± 29.64*	22
	Females	111.01 ± 34.83*	44
	Subjects with BMI<25	109.44 ± 33.35^†^	51
	Subjects with BMI>25	89.12 ± 30.71^†^	15
	Subjects with aerobic activity>150 min/wk	106.05 ± 33.88	54
	Subjects with aerobic activity<150 min/wk	99.30 ± 33.19	12
GEM-B (bridging endurance)	All subjects	81.03 ± 24.79	66
	Healthy subjects	81.68 ± 24.93	49
	Subjects with recurrent LBP	79.15 ± 25.04	17
	Males	86.61 ± 21.70	22
	Females	78.24 ± 25.98	44
	Subjects with BMI<25	83.66 ± 25.27	51
	Subjects with BMI>25	72.10 ± 19.70	15
	Subjects with aerobic activity>150 min/wk	80.95 ± 23.19	54
	Subjects with aerobic activity<150 min/wk	81.40 ± 30.14	12

* = statistically significant between sexes (p = 0.036); ^†^ = statistically significant between body mass index groups (p = 0.035); BMI = body mass index; min = minutes; N = number of subjects; s = seconds; SD = standard deviation; wk = week

Pearson’s correlation coefficients between GEM scores, MF slopes, and BMI revealed multiple significant correlations. There was a significant correlation between GEM-B scores and both gluteus maximus and gluteus medius MF slopes (r = 0.35, p = 0.004; and r = 0.44, p = 0.000 respectively), indicating lower GEM-B scores as gluteal MF slopes become steeper in the negative direction (as muscle fatigue increases). There was a significant negative correlation between BMI and gluteus maximus MF slope during GEM-B (r = -0.24, p = 0.048), indicating subjects with a larger BMI have lower GEM-B scores. Lastly, GEM-A scores showed significant but low correlation with GEM-B scores (r = 0.32, p = 0.008).

Independent t-tests revealed few statistically significant differences between sexes, BMI groups, and those with and without recurrent LBP. Scores for GEM-A were significantly lower in males than females (p = 0.036). Similarly, MF slopes for the gluteus medius were significantly steeper in the negative direction for males (-0.31 ± 0.13 Hz/s) than females (-0.17 ± 0.10 Hz/s) during GEM-A (p = 0.000), indicating higher fatigability. Scores for GEM-A were also significantly lower in subjects with a BMI greater than or equal to 25 (delineating overweight) than those with a BMI less than 25 (p = 0.035). No statistically significant differences were found between subjects with and without recurrent LBP.

Subjects reported posterolateral hip (gluteal) fatigue as the reason for task failure in 184/198 trials of GEM-A (93% of trials). Subjects reported posterolateral hip (gluteal) fatigue as the reason for task failure in 170/198 trials of GEM-B (85.86% of trials). The frequency distribution of subjective reasons for GEM-A and GEM-B failure are reported in Tables 3 and 4, respectively.

**Table 3.** Frequency distribution of subjective reasons for GEM-A failure

Subjective reason for task failure	Frequency (number; percentage) (N = 198)
Posterolateral hip (gluteal) fatigue	184; 92.93%
Contralateral posterolateral hip fatigue	7; 3.54%
Low back (erector spinae) fatigue	3; 1.52%
Anterior thigh (quadriceps) fatigue	2; 1.01%
Posterior thigh (hamstring) fatigue	1; 0.51%
Lower leg (triceps surae) fatigue	1; 0.51%

N = number of total trials

**Table 4.** Frequency distribution of subjective reasons for GEM-B failure

Subjective reason for task failure	Frequency (number; percentage) (N = 198)
Posterolateral hip (gluteal) fatigue	170; 85.86%
Posterior thigh (hamstring) fatigue	16; 8.08%
Low back (erector spinae) fatigue	12; 6.06%

N = number of total trials

## DISCUSSION

The purpose of this study was to determine the reliability and validity of two convenient, inexpensive, and unilateral gluteal endurance measures (GEMs), GEM-A (abduction endurance) and GEM-B (bridging endurance). Scores for both GEMs demonstrated high intra-rater and inter-rater reliability comparable to similar measures of core endurance such as the side bridge and supine bridge in other studies. Table 5 describes the reliability, mean scores, and SEMs of the GEMs and similar measures in adults from other studies. The SEM of GEM-A and GEM-B was lower than the SEM of similar measures in most comparable studies.^1,18,19,38–40^ Validity of the GEMs was primarily displayed with MF slope values and subjective reports of reasons for task failure.

**Table 5.** Reliability, means, and SEMs of GEMs and similar measures

Endurance measure	Intra-rater reliability (*ICC*) [95% CI]	Mean score ± SD (*s*)	SEM (*s)*
GEM-A (abduction endurance)	0.94 [0.92, 0.96]	104.8 ± 34.1	8.4
GEM-B (bridging endurance)	0.87 [0.80, 0.92]	81.0 ± 24.8	8.9
Side bridge: 1. Greene, 2012^18^ (right); (left) 2. McGill, 1999^1^ (right); (left) 3. Palmer, 2011^38^ (right); (left) 4. Waldhelm, 2012^39^ (right); (left)	0.78 [NA]; 0.91 [NA] 0.96 [NA]; 0.99 [NA] 0.91 [0.80, 0.97]; 0.89 [0.81, 0.97] 0.74 [0.30, 0.92]; 0.96 [0.87, 0.99]	75.1 ± 50.3 80.2 ± 51.4 81.0 ± 34.0 85.0 ± 36.0 70.0 ± 41.3 73.0 ± 37.0 78.5 ± 28.7 77.1 ± 37.8	23.6 15.4 6.8 3.6 12.4 12.3 14.6 7.6
Supine bridge by Shellenberg, 2007^19^	0.84 [NA]	170.4 ± 42.5	17.0
Isometric test of hip abductors with 7.5% BW load by Van Cant, 2016^40^	0.73 [NA]	88.4 ± 38.2	19.8

BW = body weight; CI = confidence interval; ICC = intraclass correlation coefficient; NA = not available; SD = standard deviation; SEM = standard error of measurement

The results of this study of GEMs indicate similarities and advantages of these measures compared to related tests. An earlier study by Van Cant et al.^40^ examined an isometric test of hip abductors with an ankle weight load equivalent to 7.5% of subjects’ body weight (BW). This is a relatively new endurance measure yet to be studied elsewhere. Among the submaximal, isometric endurance measures that are related to the gluteals and have reliability data, this test is most similar to GEM-A (abduction endurance). The positions and actions of the two measures are similar except for the external load and degree of hip abduction used during testing. The loaded test examined by Van Cant et al.^40^ employs 0° of hip abduction (with the lower extremity held above the testing surface) while GEM-A is performed with 30° of hip abduction above the testing surface. Preliminary GEM trials experimented with different angles of hip abduction. Hip abduction angles higher and lower than 30° (i.e. 45°, 15°, and 0°) resulted in hold times beyond five minutes in several subjects which would be inconvenient in the clinical setting. This may be the reason Van Cant et al.^40^ used an external load at 0° of hip abduction.

The SEM value found for GEM-A is advantageously low compared to similar measures. The 30° of abduction used and the decision not to use an external load for GEM-A appear defensible in light of comparison to the isometric test of hip abductors at 0° with a 7.5% BW load. The external load used by Van Cant et al.^40^ may be what produced notably lower reliability and SEM values for the aforementioned test at 0° (ICC = 0.73, and SEM = 19.8 seconds, respectively) compared to GEM-A (ICC = 0.94, and SEM = 8.4 seconds, respectively). An external load (i.e. an ankle weight around the ankle) is an abnormal addition to limb movement which could alter the limb’s proprioception. This may result in increased limb movement during attempted isometric contraction and less consistent hold times.

The SEM value found for GEM-B is also advantageously low compared to similar measures. The supine bridge is the endurance measure most similar in position to GEM-B (bridging endurance) with studied reliability values. Data for the supine bridge studied by Shellenberg et al.^19^ are also limited to its seminal study. It differs from GEM-B by employing the use of bilateral lower extremities and 90° of knee flexion for its starting position versus the 135° used for GEM-B. The supine bridge’s use of bilateral lower extremities may give rise to its higher SEM (17.0 seconds) compared to that of GEM-B (8.9 seconds). The mean score and standard deviation of the bilateral supine bridge (170.4 ± 42.5) were roughly twice that of GEM-B (81.0 ± 24.8). Another reason the supine bridge might have demonstrated a higher standard deviation and SEM is its lack of muscle specificity compared to GEM-B given its lower degree of knee flexion. A study of the unilateral bridge demonstrated 75% MVIC EMG activity of the biceps femoris when the knee was flexed to 90° versus 23% MVIC when it was flexed to 135°.^24^ The authors suggest this was because the lower leg is more vertically aligned when the knee is flexed to 135° (more parallel with the ground reaction force vector at the foot), so the knee extensor moment and subsequent need for hamstring activity are reduced. The lower reliability and higher SEM of the supine bridge compared to GEM-B, therefore, may be because it presents a challenge for both the hamstrings and gluteals bilaterally rather than attempting to isolate unilateral gluteal activity.

Inter-rater reliability data for other core endurance measures are similar to those of the GEMs. Larsson et al.^41^ found high inter-rater reliability for the side bridge in soldiers (ICC = 0.99). Evans et al.^42^ found similarly high inter-rater reliability for the side bridge in athletes (ICC = 0.82-0.91). Bruce et al.^43^ found high inter-rater reliability in an examination of several core muscle endurance tests (ICC = 0.99-1.00), including a dominant and non-dominant lower extremity wall sit hold and horizontal trunk hold which were likely to elicit activity from the gluteus maximus among other lower extremity and trunk muscles.^44^ Therefore, the high inter-rater reliability found for the GEMs in this study (ICC = 0.99 for GEM-A, 0.99 for GEM-B) is consistent with other submaximal isometric endurance measures related to the gluteals.

The reliability of MF slopes found in this study support their use for measuring gluteal fatigue. Reliability data for MF slopes of the gluteals during endurance tasks are limited, but slope values of the erector spinae during an extension endurance task are available for comparison. The reliability of MF slope during assessment of erector spinae fatigue via the Sorensen test (a common submaximal endurance measure for the back extensors) was shown to be high in a study by Dedering et al. (ICC = 0.70-0.87).^45^ The current study demonstrated similarly high reliability of the MF slopes of the gluteals during both GEMs (ICC = 0.70-0.83), indicating MF slope can be used as a reliable value of gluteal muscle fatigue during the GEMs.

The mean MF slopes of the erector spinae in the aforementioned study by Dedering et al.^45^ of the Sorensen test (-0.12 to -0.07 Hz/s) were similar to but flatter than the mean MF slopes of the gluteus medius and gluteus maximus in the current study during GEM-A and GEM-B (-0.22 to -0.14 Hz/s, and -0.25 to -0.08 Hz/s, respectively). This comparison indicates that the gluteals fatigue during the GEMs at a faster rate than the erector spinae fatigue during the Sorensen test. No specific slope value indicates muscle fatigue aside from a slope in the negative direction. Rather, a steeper negative MF slope generally indicates greater muscle fatigability. Median frequency slope values below zero, seen in both gluteals during both GEMs, indicate the presence of muscle fatigue.^46^

A study by Xiao et al.^34^ examined MF slopes of lower extremities during a unilateral bridge. However, 90° of knee flexion (instead of 135°) and arm placement on the ground (instead of across the chest) were used for the unilateral bridge, and gluteals were not included in the analysis. Regardless, MF slopes during the unilateral bridge with 90° of knee flexion and ground arm placement were -0.14 Hz/s for the erector spinae, -0.06 Hz/s for the rectus abdominus, and -0.12 Hz/s rectus femoris. The difference in extremity positions between the unilateral bridge used by Xiao et al.^34^ and the position of GEM-B in this study limits direct comparison, but indicates the erector spinae and other muscles may also fatigue during GEM-B. The subjective reasons for task failure during GEM-B (Table 4), however, indicate the erector spinae are not a primary source of perceived fatigue.

In addition to the negative MF slope values indicating gluteal fatigue during the GEMs, and significant gluteal MVIC EMG activity found in the positions used by the GEMs in previous studies, subjective reasons for task failure provide content validity for the GEMs.^24,47^ Of the 198 trials of GEM-A (three trials from 66 subjects), posterolateral hip (gluteal) fatigue was reported as the reason for task failure 93% of the time. Posterolateral hip (gluteal) fatigue was the reason for task failure 86% of the time for GEM-B. Both of these values demonstrate higher focus on the gluteal muscles than similar core endurance measures, including the side bridge, a modified side bridge, and the Sorensen test.^18,48^ In a study of the side bridge and a modified version with the feet elevated instead of the torso, Greene et al.^18^ asked subjects “Why did you stop the test?” immediately after each test, which is identical to the question asked to subjects following the GEM trials. Side or hip fatigue or pain was the primary reason for task failure during the side bridge in the study by Greene et al.,^18^ but it was only reported from 46% of the healthy university subjects. The primary reason for task failure during the feet-elevated version of the side bridge in the same study was higher than the traditional side bridge, reported from 68% of subjects.^18^ The most common reason for task failure in a study of the Sorensen test was reported as “fatigue” (as opposed to LBP) in 62.5% of a sample of 544 working-age men.^48^ While reasons for task failure are not reported in many studies of core endurance measures, it appears the GEMs are more specific to the gluteals than several similar measures.

Criterion validity for the GEMs remains questionable, but was provided to a small degree by the correlation between GEM scores and their respective MF slopes in the current study. There was a significant albeit low correlation between GEM-B scores and both gluteus maximus and gluteus medius MF slopes (r = 0.35, p = 0.004; and r = 0.44, p = 0.000 respectively).^49^ Correlations between GEM-A scores and MF slopes for the gluteus maximus and medius were negligible and were not statistically significant (r = 0.11, p = 0.375; and r = 0.18, p = 0.145, respectively). These results suggest that steeper negative MF slopes of the gluteals are correlated to lower GEM scores, but GEM-B scores are related to MF slope to a larger degree than GEM-A scores.

The low correlations between MF slope and GEM scores are understandable in light of studies that hypothesize reasons other than EMG variables for submaximal isometric task failure. These reasons primarily involve factors of central fatigue, including the perception of effort.^50,51^ One assumption made by attempting to measure peripheral fatigue using MF slope is that the decline in a muscle’s force generating capacity is associated to the time to task failure. It appears, from this study, that this is true to some degree, but additional mechanisms contribute to task failure of the GEMs.

There were no identified differences between measured GEM scores for healthy subjects and those with a history of recurrent LBP. This is contradictory to other studies of core endurance in subjects with LBP and does not support the current study’s hypothesis.^8,19,28^ The notable difference between this study and others, however, is the type of LBP studied. Subjects with recurrent LBP were recruited for this study while subjects with chronic LBP have been used by most studies of core endurance measures.^8,11,28^ Research does not provide much information about factors that predict recurrence in individuals who have recently recovered from an episode of LBP,^52^ which is why such subjects were examined in this study. A systematic review of the risk of recurrence of low back pain revealed a history of previous episodes of LBP was the only factor that consistently predicted recurrence of LBP.^52^ No subjects in the current study had LBP at the time of testing. So it may be that the differences in hold times seen between subjects with chronic LBP and healthy controls during the Sorensen test, supine bridge, and other core endurance measures are due to the presence of pain during testing rather than their history of low back pain. The difference may also be due to the amount of time such subjects endured their chronic LBP or its intensity compared to the subjects with recurrent LBP used in this study. Further study is needed to determine if the GEMs can be used to distinguish between healthy subjects and those with pathology, such as acute or chronic low back pain or hip pain.

Relationships between BMI and both MF slopes and GEM scores were seen in the current study. No statistically significant differences were seen between subjects with and without recurrent LBP, but BMI demonstrated a statistically significant positive correlation with gluteus maximus MF slope during GEM-B (r = 0.35, p = 0.004). Additionally, subjects with a BMI greater than or equal to 25 (delineating overweight) showed significantly lower GEM-A scores than subjects with a BMI less than 25. In a study of multiple endurance tasks (handgrip, shoulder flexion, and trunk extension exertions at varying MVIC levels), findings indicated the relationship between BMI and fatigability is task dependent.^53^ Study of the Sorensen test has found subjects with higher BMI fatigue faster during the endurance test.^8^ This is similar to the relationship between BMI and hold times seen during GEM-A.

Sex differences were also found for select GEM scores and MF slopes. Scores for GEM-A were significantly shorter in males than females, and males demonstrated higher gluteus medius fatigability as measured by MF slope during GEM-A. Sex differences during submaximal isometric core endurance measures are not consistent, but males appear to demonstrate higher fatigability of their core muscles than females.^8,23,54^ This data trend was found in the GEMs. This may be due to sex-based differences in skeletal muscles, specifically their fiber-type composition and function. There appears to be a higher proportion of slower type-I and type-IIA fibers in females versus males that mirrors lower contractile velocities found in females versus males.^55^ These sex differences may be what resulted in shorter GEM scores and higher fatigability as measured by MF slope among males compared to females in the current study.

The mean scores of the GEMs (104.83 ± 34.11 seconds for GEM-A, and 81.03 ± 24.79 seconds for GEM-B) are comparable to similar measures of core endurance and allow for convenient clinical application. Mean scores of the side bridge in healthy adults range from 77.25 to 95.42 seconds.^1,4,9,39^ Mean bilateral supine bridge scores have been reported as 170.40 seconds.^19^ Mean GEM scores between one and two minutes allow them to be performed as part of a clinical assessment without dominating the assessment time or neglecting the endurance characteristics of the gluteals. Also notable is no adverse events occurred during administration of the GEMs and the required instrumentation is minimal. So these GEMs are safe measures that can be readily utilized by clinicians. Moreover, the GEMs are simple enough to be performed and monitored by clients or athletes without clinicians.

This study is not without limitations. Surface EMG carries inherent limitations. These include electrical cross-talk between underlying muscles (causing electrodes to detect activity from muscles besides those targeted) and electrical impedance from debris on the skin. Differences in subcutaneous tissue layers can affect EMG parameters. Given these differences, a normalized (relative) MF slope calculated using the initial MF instead of an absolute measure of MF slope may have demonstrated more significant differences. Every effort was made to control for these limitations by using consistent electrode placement and cleaning and abrading the skin with isopropyl alcohol pads prior to data collection.

The GEMs were studied in male and female university students in health-related fields with and without recurrent low back pain, so the application of results to other populations is limited. This university sample between 18 and 35 years of age can be considered generally more active than adults older than 35 years, those who are less concerned with physical activity, and those with more sedentary lifestyles. Therefore, mean GEM scores found in this study are likely higher than the general population. Also, data derived from subjects with recurrent LBP in this study are limited to that location and type of pain. Areas of future study for the GEMs include subjects with chronic LBP or hip pain. Subjects with various hip pathology including femoroacetabular impingement are hypothesized to have decreased GEM scores.^56^ A prospective study would also provide more insight about the construct validity of the GEMs and risk factors for various hip or low back pathology.

## CONCLUSION

This seminal study of GEM-A (abduction endurance) and GEM-B (bridging endurance) found both measures to be reliable and valid measures of gluteal endurance. Both GEMs demonstrated high intra- and inter-rater reliability. The MF slopes of the gluteus maximus and gluteus medius during each measure also demonstrated high reliability. No significant differences were seen between subjects with and without recurrent low back pain. Validity for both GEMs was provided by the notable negative MF slopes of the gluteals during each measure. Validity was also found from the high percentage of subjects who reported posterolateral hip fatigue as the primary reason for task failure for each GEM (86-93%), and the tendency for females and subjects with lower BMI to have higher GEM scores than males and subjects with higher BMI, respectively. Further examination of the GEMs in samples with different types of LBP or hip pain is recommended. The GEMs are specific to gluteal fatigue and demonstrate low measurement error compared to similar measures. The findings of this study, therefore, allow these GEMs to be confidently used to measure gluteal endurance in clinical and athletic settings among other measures of gluteal function.

### Conflict of Interest

There are no potential conflicts of interests, including financial arrangements, organizational affiliations, or other relationships that may constitute a conflict of interest regarding the submitted work.
